# Isoquinolines from the Roots of *Thalictrum flavum* L. and Their Evaluation as Antiparasitic Compounds

**DOI:** 10.3390/molecules15096476

**Published:** 2010-09-16

**Authors:** Jacqueline Ropivia, Séverine Derbré, Caroline Rouger, Fabrice Pagniez, Patrice Le Pape, Pascal Richomme

**Affiliations:** 1 Laboratoire des Substances d’Origine Naturelle et Analogues Structuraux, UPRES-EA 921, IFR 149 QUASAV, UFR des Sciences pharmaceutiques et ingénierie de la santé, Université d’Angers, 16 Bd Daviers, 49045 Angers Cedex, France; E-Mail: pascal.richomme@univ-angers.fr (P.R.); 2 Laboratoire de Parasitologie et Mycologie médicale, BioCiT, UPRES EA 1155, Université de Nantes, Faculté de Pharmacie, 1 rue Gaston Veil, 44035 Nantes, France; E-Mail: Patrice.Le-Pape@univ-nantes.fr (P.L.P.)

**Keywords:** Antiparasitic, bisbenzylisoquinolines, isoquinoline alkaloids, Ranunculaceae, *Thalictrum flavum*

## Abstract

Alkaloids from *Thalictrum flavum* L. (Ranuculaceae) growing in the Loire valley (France) were isolated and evaluated for their antiplasmodial and leishmanicidal activities. Berberine was identified as a major component but its analogue, pseudoberberine, was isolated for the first time from this plant. As far as bisbenzylisoquinolines are concerned, thalfoetidine was also isolated and, besides, its nor- derivative, northalfoetidine, was identified as a new compound. Previously isolated alkaloids from *Thalictrum *species such as northalidasine, northalrugosidine, thaligosidine, thalicberine, thaliglucinone, preocoteine, *O*-methylcassythine and armepavine were newly described in the roots of *T. flavum*. Tertiary isoquinolines, and particularly bisbenzylisoquinolines, were found to be leishmanicidal against *L. major*. Thalfoetidine appeared as the most potent but its new nor- derivative northalfoetidine, as well as northalidasine, were of particular interest due to the fact that their potential leishmanicidal activity was not associated to a strong cytotoxicity.

## 1. Introduction

Widely distributed all over the world, *Thalictrum* species (Ranunculaceae) mainly grow in the temperate and cold zones of both hemispheres. Among secondary metabolites of these spp., isoquinoline alkaloids were the more investigated [1]. A variety of bisbenzylisoquinolines were isolated from roots and aerial parts of diverse *Thalictrum* [2]. These natural products are structurally and pharmacologically interesting since they exhibit specific antiparasitic properties [3], such as antimalarial [4,5] or antileishmanial activities [6,7,8]. *Thalictrum flavum* L., commonly known as “meadow-rue”, is a 0.5 to 1.5 m height herb growing in tallgrass meadows, ditches, marshes and other habitats at the water's edge [9]. In spite of the fact that the alkaloid contents of *T. flavum* L. originating from Balkan countries [10,11,12] or Russia [13,14] were already investigated, no pharmacological study has been reported so far.

Tropical parasitic diseases affect hundreds of millions of people, mostly in the Third World. As resistance to conventional treatments emerges, new drugs are still necessary in the treatment of protozoan parasitic diseases, such as malaria or leishmaniasis [15]. Natural products tend inherently to interact with living organisms and so are still a good source for compound leads [16]. In the present study, we describe the alkaloids of *T. flavum* L., together with their antiparasitic potential.

## 2. Results and Discussion

While complex and pharmacologically active dimeric alkaloids such as bisbenzylisoquinolines were already isolated from *T. flavum* L., structurally simpler isoquinolines were also extracted from this species [17]. The dimeric bisbenzylisoquinolines thalidasine, hernandesine and thalfoetidine were previously extracted from the roots as well from the aerial parts of this plant, whereas thalicarpine was only found in the roots and *O*-methylthalicberine only in the aerial parts. Glaucine and thalicsimidine (aporphines), thalicsine and thaliglucine (phenanthrenes), cryptopine (protopine) and thalflavine (isoquinolone) were also purified from the roots of *T. flavum* [11,13,14]. The oxoaporphines glaucine and corunnine were finally identified in the aerial parts of the plant [10,12]. Isoquinolines are structurally and pharmacologically interesting, since they exhibit specific antiparasitic properties [3] such as antimalarial [4,5] or antileishmanial activities [6,7,8]. However, no antiparasitic study for alkaloids from *T. flavum* L. was previously reported.

**Figure 1:**
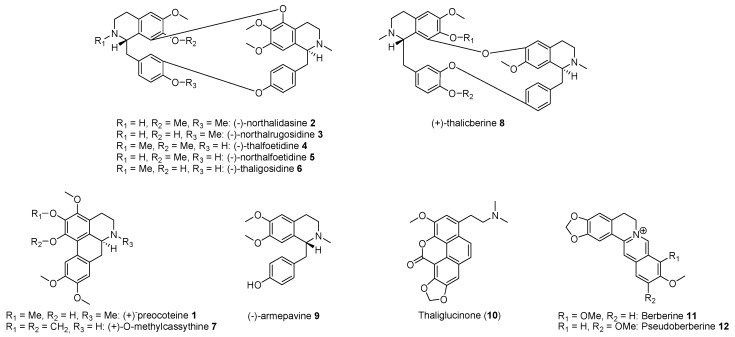
Isoquinoline alkaloids isolated from Thalictrum flavum L. roots.

In this work, as expected from a literature survey, all secondary metabolites isolated from *T. flavum* L. roots belong to the same isoquinoline group (Figure 1). As already described elsewhere, berberine (**11**) was identified as a major component [11,13]. However its analogue, pseudoberberine (**12**), was isolated for the first time from this plant. As far as bisbenzylisoquinolines are concerned, thalfoetidine (**4**) was also isolated in large amount from the roots [11] and, besides, its nor-derivative, northalfoetidine (**5**), was identified as a new compound. Previously isolated alkaloids from other *Thalictrum* species such as northalidasine (**2**), northalrugosidine (**3**), thaligosidine (**6**) and thalicberine (**8**) were characterized in the roots of *T. flavum *L. for the first time. It may be noticed that thalidasine was already isolated from underground parts [11] whereas *O*-methylthalicberine was described in aerial parts [10]. When phenanthrene derivatives were previously identified in the plant [11,13], thaliglucinone (**10**) was here identified for the first time. Finally, the aporphines preocoteine (**1**) and *O*-methylcassythine (**7**), as well as the benzylisoquinoline armepavine (**8**), were newly described in this *Thalictrum* species.

Alkaloids isolated from *T. flavum* L. (Figure 1) were evaluated in assays evaluating their antiparasitic potential as well as their cytotoxicity. As depicted in Table 1, tertiary isoquinolines from *T. flavum* L. roots, and particularly bisbenzylisoquinolines, were found to be leishmanicidal against *L. major*. Thalfoetidine (**4**) appeared as the most potent but its new nor-derivative northalfoetidine (**5**), as well as northalidasine (**2**), were of particular interest due to the fact that their potential leishmanicidal activity was not associated to a strong cytotoxicity. As far as antiplasmodial activity was concerned, the aporphine preocoteine (**1**) and the bisbenzylisoquinoline thaligosidine (**6**) were more active but ten times less potent than the reference chloroquine. As expected, quaternary protoberberines **11** and **12** appeared as antiparasitic but cytotoxic [18].

## 3. Experimental

### 3.1. General

Silica gel 60 70-230 mesh (Merck 7754, Merck Chemicals, Fontenay-sous-Bois, France) and silica gel 60 230-400 mesh (Merck 9385) were used for column and flash chromatography respectively. Tlc analyses were performed on tlc silica gel 60 F254 (Merck) with Dragendorff reagent as developing agent.

^1^H NMR, ^13^C NMR and 2D NMR spectra were recorded in the appropriate deuterated solvent on a Bruker Avance DRX 500 MHz (Bruker France, Wissembourg, France) or a Jeol GSX 270 MHz (Jeol Europe, Croissy-sur-Seine, France) spectrometer. Mass spectra were recorded on an Esquire 3000 PLUS apparatus (Bruker). The ultraviolet spectra were obtained in MeOH on a Varian Cary 50 Bio UV-vis spectrometer (Varian France, Les Ulis, France). The infrared spectra were taken on a Bruker Vector 22 apparatus (Bruker), using chloroform as solvent.

### 3.2. Plant material

The roots of *Thalictrum flavum* L. were collected in May 2005 during flowering stage along the Loire river, near Angers (France). A voucher specimen was deposited in the Musée botanique d’Angers.

### 3.3. Extraction and isolation

Alkaloids of the air-dried and powdered roots of *Thalictrum flavum* L. were extracted following a classical protocol. Briefly, the plant material (300 g) was successively extracted with CH_2_Cl_2_/MeOH (1:1, 3 L), MeOH (3 L) and water (3 L). Organic layers were combined and evaporated under reduced pressure to yield 23.7 g of crude extract. This extract was solubilised in an acidic aqueous phase (HCl 0.1 N) then successively washed with cyclohexane and CH_2_Cl_2_. The aqueous phase was subsequently alkalinised using NH_4_OH, then extracted with CHCl_3_ to give 1.63 g of tertiary alkaloids.

This alkaloid extract (1.63 g) was subjected to a vacuum liquid chromatography (VLC) over silica gel using a gradient elution (CH_2_Cl_2_ to CH_2_Cl_2_/MeOH (8:2) to yield 12 fractions (A-L).

Fraction C (58 mg) was purified using a medium pressure liquid chromatography (MPLC) over Si gel [CHCl_3_/MeOH/NH_4_OH (95:5:0.2)] to give 6 mg of (+)-preocoteine (**1**) [19,20].

Fraction E (191 mg) was subjected to MPLC over Si gel [hexane/EtOAc/DEA/MeOH (74:20:5:1)] to afford 7 mg of (*-*)-northalidasine (**2**) [21], 7 mg of (*-*)-northalrugosidine (**3**) [22], 29 mg of (*-*)-thalfoetidine (**4**) [2,23] as well as 10 mg of a new compound (**5**). Fractions 16-22 (20 mg) were subjected to preparative TLC using the same eluent to give 10 mg of **4**, 4 mg of (*-*)-thaligosidine (**6**) [24] and 2 mg of (+)-*O*-methylcassythine (**7**) [25,26].

Fraction F (432 mg) was purified using a MPLC over Si gel [hexane/EtOAc/DEA/MeOH (73:20:5:2)] to give 11 mg of **4**, 7 mg of 5 and 53 mg of (+)-thalicberine (**8**) [27]. Fractions F-9-15 (80 mg) were also subjected to a MPLC, [cyclohexane/EtOAc/MeOH/DEA (90:5:5:5)] to give 3 mg of **5**, 16 mg of **6** and 3 mg of (*-*)-armepavine (**9**) [28]. Fractions F-20-34 (110 mg) were purified using MPLC with cyclohexane/EtOAc/MeOH/DEA (70:20:5:5) to give 24 mg of (**8**) and 6 mg of thaliglucinone (**10**) [29,30].

Fraction H (208 mg) was finally purified through MPLC (Si gel) using cyclohexane/CH_2_Cl_2_/DEA/MeOH (70:20:5:5) as the eluent to give 22 mg of berberine (**11**) [18] and 51 mg of pseudoberberine (**12**) [31,32].

**Table 1:** *In vitro* cytotoxicities and antiparasitic activities of compounds **1**-**12**.

Biological activities	Cytotoxicity				Antimalarial IC_50_		Leishmanicidal IC_50_	
	MCR-5 cells		KB cells		*Plasmodium falciparum* FcB1 Colombia		*Leishmania major*	
	10 µg/mL	1 µg/mL	10 µg/mL	1 µg/mL	µg/mL	µM	µg/mL	µM
(*+*)-Preocoteine **1**	20		23		0.5	1.3	>100	
(*+*)-*O*-Methylcassythine **7**	61	4	49		3.1	8.6	ND	
(*-*)-Armepavine **9**	0		0		3.6	11.4	ND	
(-)-Northalidasine **2**	0		32		3.4	5.2	27	41
(-)-Northalrugosidine **3**	77	0	51	0	2.7	4.3	30	48
(-)-Thalfoetidine **4**	62	0	40		2.1	3.3	17	27
(-)-Northalfoetidine **5**	6		44		2.8	4.5	39	63
(-)-Thaligosidine **6**	61	0	47		1.2	2.0	38	61
(*+*)-Thalicberine **8**	29		43		2.5	4.2	55	90
Thaliglucinone **10**	79	21	76	24	2.4	6.7	63	173
Berberine **11**	ND		ND		0.4	0.9	13	35
Pseudoberberine **12**	ND		ND		0.5	1.3	3.5	9
Chloroquine					0.06	0.1		
Pentamidine							28	82

Compound **5** was identified as a new nor-derivative, *(-)-northalfoetidine*: Rf 0.35 (hexane/EtOAc/DEA/MeOH 70:20:5:5). UV (MeOH) λmax (nm): 275. ^1^H-NMR (500 MHz, CDCl_3_): 7.52 (1 H, dd, *J *= 8.5, 2 Hz, H-14’), 6.97 (1 H, dd, *J* = 8.5, 2 Hz, H-13’), 6.81 (2 H, d, *J *= 9 Hz, H-13, H-14), 6.48 (1 H, dd, *J* = 8.5, 2 Hz, H-11’), 6.45 (1 H, s, H- 8’), 6.34 (1 H, dd, *J* = 8.5, 2 Hz, H-10’), 6.30 (1 H, s, H-5), 6.28 (1 H, d, *J* = 2 Hz, H-10), 3.89 (2 H, *m*, H-1, H-1’), 3.87 (3 H, s, 6’-OMe), 3.76 (3 H, s, 6-OMe), 3.47 (3 H, s, 7-OMe), 3.27 (3 H, s, 7’-OMe), 3.23 (2 H, *m*, H- α, H- α’), 2.90 (2 H, *m*, H-3, H- 3’), 2.80 (2 H, *m*, H- α, H- α’), 2.65 (3 H, s, N’-Me), 2.40 (2 H, *m*, H-4, H- 4’), 2.35 (2 H, *m*, H-3, H- 3’), 2.15 (2 H, *m*, H-4, H- 4’). ^13^C-NMR (125 MHz, CDCl_3_): 153.5 (C-12’), 152.0 (C-6), 151.0 (C-6’), 147.7 (C-11 or C-12), 147.1 (C-11 or C-12), 143.8 (C-8, C-5’), 137.8 (C-7), 137.6 (C-7’), 134.2 (C-9, C-9’), 132.5 (C-14’), 132.2 (C-4a'), 131.2 (C-10’), 124.0 (C-8a), 121.6 (C-4a), 119.4 (C-13’), 119.2 (C-11’), 114.9 (C-13, C-14), 114.3 (C-10), 106.1 (C-8’), 105.4 (C-5), 64.9 (C-1, C-1’), 60.5 (7’-OMe), 60.2 (7-OMe), 56.2 (6’-OMe), 55.8 (6-OMe), 51.2 (C-3, C-3’), 43.9 (N’-Me), 40.8 (C-α, C-α’), 24.1 (C-4, C-4’). HREIMS: *m/z* 625.2908 (calcd for C_37_H_41_N_2_O_7_^+^, 625.2909). [α]_D_^20°C^: - 27 (c = 0.07, CHCl_3_).

### 3.3. Evaluations of cytotoxicity towards MCR-5 and KB cells

Cytotoxicities were evaluated at the ICSN-CNRS (Gif sur Yvette, France) on MRC5 and KB cells, in DMSO, at 10 and 1 µg/mL according to the procedure described by Moret *et al. * [33].

### 3.4. In vitro P. falciparum culture and activity assays

Antimalarial activity was evaluated on FcB1 Colombia strain of *Plasmodium falciparum* according to a previously described procedure [34].

### 3.5. In vitro leishmanicidal activity

*Leishmania major* (MHOM/Il/81/BNI) was cultivated at 26 ºC in a Schneider’s insect medium (Sigma, St Quentin Fallavier, France) supplemented with 15% foetal bovine serum (FBS) (Sigma), penicillin (100 IU/mL) and streptomycin (50 µg/mL). Exponentially growing cells were maintained at 26 ºC. Promastigote susceptibility testing was performed with the Uptiblue® micromethod previously described [35]. Briefly, 100 µL of a 106 promastigotes/mL suspension were placed into wells of a 96-well microplate (Nunc®). The cultures were exposed for 96 h at 26 ºC to the antileishmanial drugs at the concentrations used above, except for meglumine antimoniate for which dilutions were 0.2, 2 and 20 mg·mL^-1^. Four h before measurement, 10 µL of Uptiblue® were added. The fluorescence was measured at 590 nm with an excitation at 550 nm.

## 4. Conclusion

In summary, twelve isoquinolines were isolated from the roots of *Thalictrum flavum* L. As expected, berberine **11** was identified as the major component but its analogue, pseudoberberine **12**, was isolated for the first time from this plant. Six bisbenzylisoquinolines (**2**-**6** and **8**) were identified: As previously described, thalfoetidine **4** was isolated in large amount but, more interesting, its nor- derivative, northalfoetidine **5**, was identified as a new compound. Moreover, bisbenzylisoquinoline, aporphine, phenanthrene and benzylisoquinoline alkaloids, previously isolated from *Thalictrum *species, were newly described in the roots of *T. flavum*. This chemical composition is slightly different from those previously described. This could be due to either seasonal variations or a difference in the soil type. Indeed, previous studies were on *T. flavum* from Balkan countries or Russia, while we worked on a sample originating from the Loire valley. As far as antiparasitic activity was concerned, tertiary isoquinolines, especially bisbenzylisoquinolines, were found to be leishmanicidal against *L. major*. Thalfoetidine **4** appeared as the most potent but its nor- derivative northalfoetidine **5**, as well as northalidasine **2**, were of particular interest as their potential leishmanicidal activity was not associated to a strong cytotoxicity.
